# Spatial and temporal dynamics in the use of urban habitats by Hooded Crows

**DOI:** 10.1038/s41598-026-40561-z

**Published:** 2026-02-19

**Authors:** Petra Paládi, Isma Benmazouz, Máté Tóth, László Kövér, Szabolcs Lengyel

**Affiliations:** 1 HUN-REN Centre for Ecological Research, Institute of Aquatic Ecology, Conservation Ecology Research Group, Bem tér 18/c, Debrecen, 4026 Hungary; 2 Department of Nature Conservation, Zoology and Wildlife Management, University of Debrecen, Böszörményi út 138, Debrecen, 4032 Hungary; 3 Department of Metagenomics, University of Debrecen, Nagyerdei körút 98, Debrecen, 4032 Hungary; 4 Biodiversity, Water Management and Climate Change Competence Centre, University of Debrecen, Vezér utca 37, Debrecen, 4032 Hungary

**Keywords:** nuisance species, urban biodiversity, urban ecology, urban wildlife management, urbanization, Corvidae, Ecology, Urban ecology

## Abstract

Understanding the habitat use of animals in cities is relevant for urban planning, human-wildlife conflict management and urban biodiversity conservation. We studied the habitat use of Hooded Crows, a nuisance bird in much of Europe, in 16 different neighborhoods (sections) of a recently crow-colonized city in E Hungary. In the breeding season, when crows defend territories, crow numbers increased with nesting site availability and the number of trashbins but were unaffected by habitat type or area of the section. However, colonization probability was negatively influenced by the number of trashbins and restaurants, was high in parks, sports complexes, and quieter residential areas, and was low in residential areas busy with traffic. Outside the breeding season, when crows move around in groups, crow numbers increased with time in the section with the highest number of trashbins, decreased in residential areas and was stable in parks and sports complexes. Our results suggest that while crows are attracted by the foraging opportunities offered by anthropogenic food sources, they avoid nesting in such areas, likely due to high human disturbance. This implies that residential areas with fewer food sources will attract fewer crows, potentially reducing human-crow conflicts. Our study also suggests that improved waste management, such as closed-top trashbins in public places or covered enclosures in zoos, may further reduce the availability of anthropogenic food sources to crows and that targeted crow control is best scheduled for the breeding season and in residential areas.

**Supplementary Information:**

The online version contains supplementary material available at 10.1038/s41598-026-40561-z.

## Introduction

Urbanization involves the spatial expansion of urban habitats and the intensification of urban use of already built-up areas, both of which have increased rapidly in recent decades^1^. Novel urban habitats cause changes in the composition of species communities^2^. The species richness of birds usually decreases in urban areas^3,4^, and urbanization leads to a biotic homogenization of the communities^5,6^. This is often accompanied by a decrease in functional diversity in cities^7^.

Living in cities causes some species to change rapidly, often within a few generations. This accelerated evolution can be observed in cities globally, in species of insects, lizards, or even birds^8^. The adaptation process of wild animals to urban areas is often termed as synurbization^9^, and species that are found in urban habitats are synurbic species^10^. However, this term is not yet fully clarified, as some authors consider a species as synurbic only if it has higher density in urban areas than in rural ones^11^. In birds, omnivorous or granivorous species, and cavity nesters are more likely to develop adaptations to urban habitats^12^.

Urban areas affect species in different ways, both negatively and positively^13,14^. One main negative factor is direct disturbance by humans^15^, and indirect disturbance by roads and traffic^16^. Birds must adapt to the different microclimatic conditions, which can alter their nesting characteristics and success^17–19^. Additional negative effects are lack of suitable nesting sites, lower food quality and body condition^20,21^, spread of diseases and parasites, and fatal collisions with cars or buildings^12^. Light pollution changes the natural circadian cycle and deteriorates the sleep cycle, and prolonged diurnal activity may alter mating behaviour and reproductive success^22^. Noise pollution can also impact the vocalization, communication, space use and daily rhythm of birds^23^. Urban birds are also exposed to direct and indirect effects of chemical pollution^24^.

Urban areas can also offer benefits to some species. Urban areas usually provide higher chances for survival and more efficient breeding than rural areas^25,26^. Mortality due to predation can be lower in urban areas than in rural ones^27,28^, and urban species can develop resistance against certain parasites^29^. Through the heat island effect, cities can offer better conditions for conserving metabolic energy, resulting in lower costs of thermoregulation, and eventually higher survival than rural areas^30^. Urban areas can offer diverse nesting opportunities and food sources^28^. Despite these benefits, the exact causes of synurbization are rarely known and the relative roles of the ecological flexibility and the phenotypic plasticity of species are often debated^31^.

At least 30 species of corvids (*Corvidae* family, c. 139 species) are found regularly in urbanized areas^32^. Many of the species are ‘urban exploiters’, that can live and forage fearlessly among humans, are highly social, and can exploit anthropogenic resources^33^. Species with these traits are widespread breeders in many cities around the world^34–38^. Corvids have long been known to occur in cities. There is evidence that their numbers decreased in the 18th and 19th centuries, however, since the end of the 19th century, population growth was perceptible in both the number of species and the density of populations in cities^28,39^. Corvids can utilize a wide range of food sources^40,41^. They are highly intelligent and adaptable^42–44^ and have the ability of social learning^45^, which means that the behavioural changes in an animal are shaped by various social interactions. Corvids can also pass on knowledge to their offspring and their conspecifics through social learning^46^, which greatly facilitates adaptation to the new urban environment, for example, through learning to avoid potential threats^47^.

The Hooded Crow (*Corvus cornix*) is a large corvid species widespread in cities in Europe^36,48^. This species was typical in rural areas with arable lands^49^ and has colonized cities in eastern and northern Europe several decades ago^28,50^, and cities in central Europe more recently^37^. Several hypotheses explain the synurbization of Hooded Crows^51^ but most authors focus on nest site availability, food availability and predation pressure. First, urban Hooded Crows can exploit nesting resources not available in rural areas such as tall trees^37^, buildings^52^ or high-voltage pylons^53^. Second, the availability of food can also be higher in cities^54^, although its quality can be lower than in rural areas^55^, and Hooded Crows can exploit diverse food sources. Most typically, they hunt for invertebrates in grass lawns but can also hunt for fish, amphibians or small mammals. They supplement their diet with urban pet food, zoo animal food or food from trashcans and landfills^56^, and road-killed animal carcasses^57^. Finally, predation and hunting pressure may be lower in cities than in rural areas, where hunters or farmers often persecute crows. The main predator of Hooded Crows, the Eurasian Goshawk (*Accipiter gentilis*), rarely hunts in urban areas, and hunting pressure from humans does not exist in cities.

The space use of urban Hooded Crows differs considerably between the breeding season and the non-breeding season. In the breeding season, Hooded Crows’ presence is positively correlated with suburban green areas, bare soil and grass cover^58^ and their density is positively influenced by parks, green spaces and tall trees, and negatively by dense tree cover^37,59^. In the breeding season, nesting and chick-rearing pairs are territorial, have small home ranges and try to chase conspecifics away from the territory. In contrast, after the breeding season, families with fledged young aggregate and typically move together in groups of up to 200–300 birds in the winter (e.g.^36,60^. Interestingly, we know very little about habitat use of Hooded Crows in urban environments. While several studies investigated urban habitat use in the Eurasian Magpie (*Pica pica*)^61–67^ and two studies were conducted in other species (Common Raven  (*Corvus corax*)^68^, Western Jackdaw (*Coloeus monedula*))^69^, the urban habitat use of Hooded Crows has not yet been compared across habitat types or between the breeding and non-breeding seasons. Understanding the habitat use of animals in urban environments is interesting not only from natural history, community ecology and evolutionary biology but also for urban planning, human-wildlife conflicts and biodiversity conservation.

The aim of our study was to better understand the factors influencing habitat use and the colonization-extinction dynamics of Hooded Crows in different parts (sections) of a recently colonized urban environment. We first used a repeated-measures analysis to test the effects of habitat type (parks, residential areas, sports complexes) and habitat quality (patch area, availability of anthropogenic food, and availability of nesting sites) on the number of crows recorded in the city sections both in the breeding season, when crows are territorial and sedentary, and outside the breeding season, when crows are gregarious and move around a lot. We used the number of restaurants and trashbins to characterize anthropogenic food availability and the number of nests as a proxy to characterize nest site availability in and near each section. We specifically asked how the number of crows varies by date and location, whether crow habitat use differs between habitat types and whether higher-quality sections (larger area, more food, more nest sites) will be used by more crows. Second, we applied multi-season and multi-site occupancy modelling to assess the colonization-extinction dynamics of the sections between breeding seasons and to assess if there are hotspots of colonization or extinction within the city across the years. We used data from an intensive survey of crows implemented once every few days for three years in the 16 city sections in both analyses.

## Methods

### Study area and field survey

**Fig. 1:**
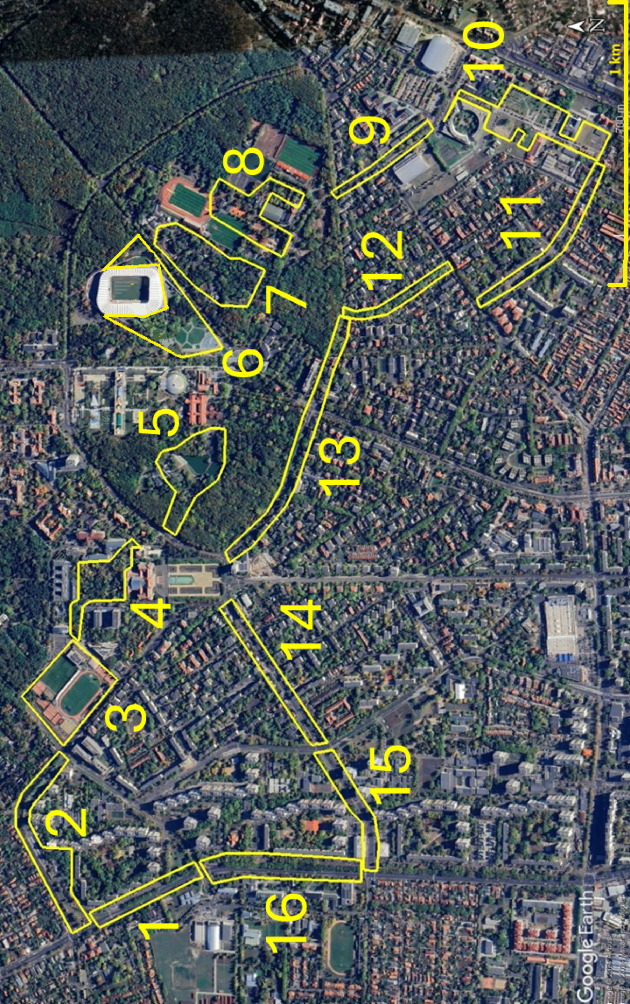
The 16 study sections (yellow polygons) in the northern part of the city of Debrecen (Numbers correspond to the ID column in Table 1. Source: Google Earth).

The study area was in the northern part of the city of Debrecen in eastern Hungary (Fig. 1, Tables 1, 47°33’07” N, 21°37’35” E). This area was the first to be colonized by Hooded Crows in the city in the early 2000s. Between 2006 and 2012, the number of pairs nesting in the city increased exponentially from around 10 to above 75 pairs^37^, and it further increased to c. 150 pairs until 2019^70^.

For this study, we designated 16 city sections for detailed surveys along a transect (length c. 10 km) in northern Debrecen (Fig. 1). The transect encompassed most of the areas that are regularly used by Hooded Crows in high numbers both in the breeding and the non-breeding seasons. During breeding, crows are territorial and nest in highest density in the areas covered by the transect^37^. Outside breeding, crows from all parts of the city aggregate in flocks that move around in the areas surveyed, resulting in very few crows in other parts of the city. The 16 sections represented main urban habitat types (residential, recreational, mixed-use) and differed in area and availability of anthropogenic food sources and nesting sites (Table 1). Because the transect was designated to maximize the chances of observing Hooded Crows both in the breeding and the non-breeding periods, the surveyed sections are not a random sample of all possible city sections, and are thus representative of city parts frequented by Hooded Crows (such as areas near zoos^71^, but not of the ‘average’ city section.

**Table 1:** Main characteristics of study sections in Debrecen city (The number of trashbins, restaurants, and nests are given for the section area plus the small (100 m) and the large (200 m) buffers.

ID	Section	Area (ha)	Habitat type	No. trashbins		No. restaurants		No. nests	
				100-m	200-m	100-m	200-m	100-m	200-m
1	Böszörményi St. N	2.0	residential	23	37	3	10	1.0	4.5
2	Doberdó St.	4.0	residential	27	36	4	8	2.5	5.0
3	DEAC Complex	5.0	sports complex	14	40	2	7	1.0	3.5
4	Egyetem Square	2.6	park	56	82	18	21	1.0	4.0
5	Békás Lake	3.8	park	72	100	8	18	4.0	5.5
6	Stadium	4.5	sports complex	33	42	3	14	3.5	6.5
7	Zoo	4.5	park	17	49	3	6	6.0	7.0
8	Oláh G. Complex	3.5	sports complex	16	23	3	3	3.0	8.5
9	Martinovics St.	1.0	residential	5	28	4	4	1.0	3.5
10	Kassai Campus	3.8	park	34	39	3	4	1.0	3.0
11	Laktanya St.	2.0	residential	5	9	4	5	0.5	2.0
12	Gvadányi St.	1.0	residential	2	9	0	2	2.5	2.5
13	Nagyerdei Blvd.	3.5	park	21	47	1	9	3.5	8.0
14	Bolyai St. East	2.5	residential	14	32	8	9	1.5	4.5
15	Bolyai St. West	2.0	residential	15	28	2	6	1.0	3.0
16	Böszörményi St. S	3.6	residential	23	44	7	10	3.0	5.5

For number of nests, means from two years are given). While population surveys were conducted throughout all three study years (2017–2019), detailed nest surveys were performed only during two of these years (2017 and 2019); therefore, the mean nest values presented in Table 1 refer specifically to the years when nest data were available.

We surveyed Hooded Crows in the 16 study sections for three years between December 1, 2016, and December 31, 2019, completing a total of 241 surveys during this period. The surveys covered both the winter season (December–February) and the breeding season (March–July) each year. Surveys were conducted once or twice weekly, with one visit per survey day, resulting in 6.5 surveys per month on average and in approximately 80 surveys per year. The surveys were carried out by a single observer (PP) on foot along the 10-km transect. Observations were performed between 10:00 and 16:00. Although starting times varied slightly depending on daylight and weather conditions, this time frame was consistently applied throughout all years to ensure data comparability. During each survey, the observer recorded the exact time, location, and number of Hooded Crows observed in each section. While we could not exclude the possibility of double-counting of individuals moving between the surveyed sections during the survey, its probability was small. In the breeding season, crows are territorial and pairs do not move much outside their territories. In the non-breeding season, crows moved around in flocks, which were relatively easy to identify and follow during the surveys and which were recorded only where they were first seen. The primary response variable in the analyses was the number of Hooded Crows recorded (for the repeated-measures analysis) or the presence or absence of Hooded Crows (for the occupancy modelling) within a given study section during one survey.

### Variables

We studied the effects of habitat type, area, anthropogenic food availability and nesting site availability on the number and occupancy of Hooded Crows in the different sections. We selected three habitat types: (i) parks, where the first nests within the city were observed and which still serve as centers for nesting^37^, (ii) residential areas with tree rows, where conflicts between humans and crows mostly occur, and (iii) sports complexes, which provide large grassy areas for crow foraging.

We measured the area of each study section (habitat patch) in Google Earth (Fig. 1), and we used patch area to specifically model the possible effect of habitat area on crow numbers, based on the hypothesis that more crows can be expected in larger patches of habitat. Because crows also use resources outside the surveyed study sections, we considered the buffer zones around the sections in the estimation of resource availability. We designated two buffer zones, one with 100 m and the other with 200 m from the borders of the polygons enclosing the study sections, to allow the analysis of possible scale-dependence of parameters influencing habitat use. These buffers also corresponded to the average size of the home range of Hooded Crows in Debrecen, which was estimated in a previous study at 19.9 ± 32.80 ha (*n* = 12, range 3.1 ha in summer to 35.1 ha in winter)^72^.

To quantify the availability of anthropogenic food sources, we surveyed and recorded the coordinates of the number of trashbins and the number of restaurants in the study sections and in their buffer zones. Trashbins and restaurants (including food-stalls, kiosks etc.) provide almost constant sources of food to crows, resulting in no need to search for food, which may determine their habitat use^50^. For the analysis, we used the number of trashbins or restaurants in the section area plus the 100-m or the 200-m buffer zone (Table 1).

To quantify nesting site availability, we used data from a detailed survey of active nests of Hooded Crows, which was implemented in the northern part of Debrecen in 2018 and 2019^70^. In this survey, we mapped active nests in a grid-based design from early March to late May. Because more detailed data such as a tree inventory with tree characteristics (species, height etc.) were not available to estimate nest site preference, we assumed that the number of Hooded Crow nests in an area reflected its suitability for nesting and thus calculated the number of nests averaged for the two years in the section and its buffer as a proxy for nest site suitability (Table 1).

### Statistical analyses

We implemented two analyses. In the first analysis, we used repeated measures ANOVA to test whether the number of crows observed on one survey occasion (response variable) depended on habitat type (parks, residential areas, sports complexes) or habitat quality (area, number of restaurants, trashbins, nests). In this analysis, the within-subject factor was time (measured by Julian date, i.e., the number of days since the start of the surveys), the between-subject factor was habitat type, and covariates were habitat area, the number of trashbins, the number of restaurants and the number of nests in the sections and their buffer zones. For the repeated-measures analysis, we used the ‘anova_test’ function of R package ‘Rstatix’^73^, which provides a pipe-friendly framework for different types of ANOVA tests including repeated-measures ANOVA by applying a wrapper around R functions ‘Anova()’ and ‘aov()’ (https://www.datanovia.com/en/lessons/repeated-measures-anova-in-r/). We checked whether the assumptions of the repeated-measures analysis were met by using the Shapiro-Wilks test for normality (‘shapiro_test’ function) and by visual inspection of boxplots to identify outliers (‘identify_outliers’ function). Because the number of crows was not normally distributed, we log-transformed the response variable for analyses. One important assumption of repeated-measures ANOVA models is that the variances of the differences between all combinations of related groups are equal (assumption of sphericity). To avoid biases, we applied the Greenhouse-Geisser sphericity correction to within-subject factors violating the sphericity assumption (‘get_anova_table’ function) in all analyses. We chose this correction as it is more conservative than the Huynh-Feldt correction, which yields better power when violations to the assumption are milder^74,75^.

We performed one analysis with the 100-m buffer zones and another with the 200-m buffer zones. Moreover, because Hooded Crows are territorial in the breeding season but gregarious outside the breeding season, we performed these analyses separately for these two periods. Based on our previous observations in the Debrecen population of Hooded Crows (PALÁDI et al. unpublished results), we considered the period between April 1 and May 31 as the breeding season, and the period from June 1 to March 31 as the non-breeding season. Finally, to allow the study of changes with time, we included all interactions between covariates on one side and Julian date on the other. In each of the four cases (two buffer sizes, two time periods), we started with the full model and removed non-significant between-subject effects, covariates or interaction terms in a stepwise approach to obtain a minimum adequate model, which we used to estimate generalized effect sizes. Terms involved in significant interactions were not removed even if they were not significant independently.

In the second analysis, we used multi-season, multi-site occupancy modelling to study the colonization-extinction dynamics of Hooded Crows across the breeding seasons in the 16 city sections. These occupancy models enable the calculation of colonization and extinction probabilities, as well as the estimation of initial occupancy and detection probability for each study section. Parameters can be modelled as functions of covariates^76^. These models assume that the study population is closed in the study periods^76^. To meet this assumption, we used data only from the breeding season (April 1 to June 30 each year) and assumed that the crow population of the study area closed in this period. This was a reasonable assumption because Hooded Crows are highly territorial in the breeding season (nesting and chick-rearing), resulting in little immigration or emigration of individuals in this period.

We used the ‘colext’ function of the ‘unmarked’ package of R to estimate models of occupancy^77,78^. We first assumed that the initial occupancy and detection probability will depend on habitat type and area only and that the other covariates (number of trashbins, restaurants, nests) do not influence initial occupancy or detection probability. We then progressively added one covariate in a forward stepwise logic to explain colonization and extinction probabilities. In the most complex model, colonization and extinction probabilities each were modelled as functions of habitat type and area, and the number of trashbins, restaurants, and nests in the section and its buffer. We ran models with both the 100-m and the 200-m buffers. We then compared the models fit with the ‘fitList’ and ‘modSel’ functions, which calculated Akaike’s Information Criterion corrected for low sample sizes (AICc) and we accepted models with ΔAICc < 2 from the best model (lowest AICc) as the final models. We then calculated probabilities of initial occupancy, colonization, extinction and detection by model-averaged estimates from these final accepted models for each site. We then built general linear models (function ‘lm’ in package ‘stats’) to test whether the probabilities differed between habitat types or varied with habitat area, number of trashbins, restaurants, and nests. For each probability, we first fit the full model and then eliminated non-significant terms to obtain the minimum adequate model, which we used for testing the significance of parameters. We checked possible deviations from normality, skewness and presence of outliers by checking the residuals in built-in diagnostic plots obtained by ‘plot(model)’.

We used Quantum GIS (version 3.36.0) to calculate buffers and count the number of trashbins, restaurants, and nests in the sections plus their buffers. For all statistical analyses, we used the R statistical environment (version 2024.04.2; R CORE TEAM, 2024).

## Results

### Sample size

We conducted 241 surveys of Hooded Crows in the 16 study sections in three years during which we walked more than 2650 km. In these surveys, we recorded 7144 observations of Hooded Crows. Two sections had large numbers of crows (Zoo: 4054 observations, Békás pond: 1719 observations). The average number of crows observed per hectare per occasion ranged from 0 (Bolyai St., Böszörményi St. residential areas) to maximum values of 1.9 (Békás pond) and 3.7 (Zoo). The median number of crows per hectare per occasion was highest in sports complexes (median 0.4, Q1-Q3: 0.1 to 0.5, *n* = 3), followed by parks (0.1, 0.06 to 2.8, *n* = 5), and was lowest in residential areas (0.01, 0 to 0.1, *n* = 8) (Kruskal-Wallis H = 6.102, *p* = 0.045).

### Factors influencing the number of crows in a city section throughout the year

**Fig. 2:**
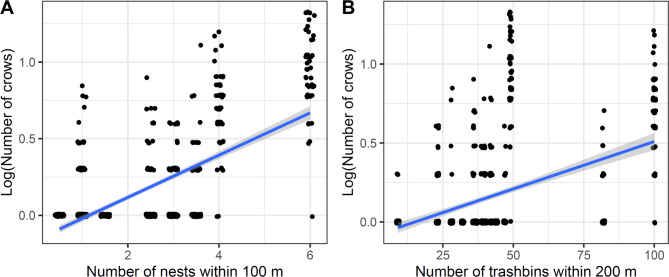
The number of Hooded Crows observed in the breeding season (April 1 to June 30) in 16 urban sites as a function of the number of crow nests in the small buffer (100 m) and the number of trashbins in the large buffer (200 m).

The repeated-measures analysis showed that in the breeding period, the number of observed crows increased with the number of nests in the small (100 m) buffer and depended on significant interactions between the number of trashbins and Julian date and the number of nests and Julian date (Table 2; Fig. 2, Fig. S1A,C). In the large buffer (200 m), crow numbers increased with the number of trashbins and were influenced by interactions between Julian date on one side, and number of trashbins, restaurants, and nests on the other (Table 2; Fig. 2, Fig. S1E,G,I). The interactions arose mainly because crow numbers increased with time in a few sections with many trashbins, restaurants, and nests, and where there were more crows initially, whereas crow numbers were stable or decreased in other sections (Fig. S1). Habitat type and area were not part of the final models in any of the four cases.

**Table 2:** Minimum adequate models of repeated-measures analyses of variance testing the effects of habitat type, area, number of trashbins, number of restaurants and number of nests on the number of crows in 16 sections of the city of Debrecen (Significant (*p* < 0.05) effects are highlighted in bold).

Season	Buffer size	Main effect or interaction	Generalized effect size (partial eta)	F	df_num_, df_denom_	*p*
Breeding	100 m	N trashbins	0.075	1.939	1,13	0.187
		**N nests**	**0.450**	**19.456**	**1**,**13**	**0.0007**
		Julian date	0.039	1.168	50,650	0.205
		N trashbins * Julian date	**0.060**	**1.820**	**50**,**650**	**0.0007**
		N nests * Julian date	**0.043**	**2.180**	**50**,**650**	**< 0.0001**
	200 m	**N trashbins**	**0.319**	**8.756**	**1**,**12**	**0.012**
		N restaurants	0.202	4.718	1,12	0.051
		N nests	0.063	1.262	1,12	0.283
		Julian date	0.020	0.671	50,600	0.960
		N trashbins * Julian date	**0.043**	**1.521**	**50**,**600**	**0.014**
		N restaurants * Julian date	**0.048**	**1.685**	**50**,**600**	**0.003**
		N nests * Julian date	**0.043**	**1.521**	**50**,**600**	**0.014**
Non-breeding	100 m	N trashbins	0.140	3.109	1,13	0.101
		N restaurants	0.051	1.020	1,13	0.331
		Julian date	0.026	1.094	189,2457	0.189
		N trashbins * Julian date	**0.050**	**2.149**	**189**,**2457**	**< 0.0001**
		N restaurants * Julian date	0.019	0.784	189,2457	0.985
	200 m	Habitat type	0.003	0.024	2,10	0.976
		N trashbins	0.145	2.507	1,10	0.144
		N restaurants	0.133	2.261	1,10	0.164
		N nests	0.049	0.761	1,10	0.403
		Julian date	0.029	0.920	189,1890	0.769
		Habitat type * Julian date	**0.071**	**1.178**	**378**,**1890**	**0.017**
		N trashbins * Julian date	**0.047**	**1.518**	**189**,**1890**	**< 0.0001**
		N restaurants * Julian date	**0.041**	**1.304**	**189**,**1890**	**0.005**
		N nests * Julian date	**0.044**	**1.414**	**189**,**1890**	**0.0003**

Outside the breeding season, none of the covariates influenced the number of crows independently of time. A significant interaction between Julian date and number of trashbins in the small (100 m) buffer (Table 2) was because crow numbers increased only in the section with the highest number of trashbins (Békás pond) and decreased or were stable in all other sections (Fig. S1B). In the large buffer, a significant interaction between habitat type and Julian date indicated that the number of crows decreased in residential areas and remained stable in parks and sports complexes (Fig. S1D). Further significant interactions arose because crow numbers decreased with time in the Zoo but did not change in the other sections (Fig. S1F,H,J). Again, neither habitat type nor area influenced the above patterns as these variables were not part of the final models.

### Colonization-extinction dynamics in the breeding season

The multi-season occupancy modelling showed that there were three final models (ΔAICc < 2) (Table 3). The best models indicated that the number of trashbins, restaurants, and nests each were important in explaining the colonization-extinction dynamics of the surveyed sections, while habitat type and area played little role. The ranking of the models showed that the number of trashbins and restaurants appeared primarily important of these covariates, followed by the number of nests, and that these relationships were stronger at the small buffer scale (100 m) than at the large buffer scale (200 m) (Table 3).

**Table 3:** Comparison of occupancy models testing the effects of habitat type (H), habitat area (A), and the number of trashbins (T), number of restaurants (R) and number of nests (N) in buffers of 100 m (T1, R1, N1) and 200 m (T2, R2, N2) in and surrounding 16 sections in the city of Debrecen.

Model	*N* of parameters	AICc	ΔAICc	AICc weight	Cumulative AICc weight
psi(HA)gam(T1R1)eps(T1R1)p(HA)	14	686.23	0.00	0.39	0.39
psi(HA)gam(T1R1N1)eps(T1R1N1)p(HA)	16	686.97	0.74	0.27	0.66
psi(HA)gam(T2R2N2)eps(T2R2N2)p(HA)	16	687.42	1.19	0.22	0.88
psi(HA)gam(T2)eps(T2)p(HA)	12	690.27	4.04	0.052	0.93
psi(HA)gam(T2R2)eps(T2R2)p(HA)	14	690.66	4.42	0.043	0.97
psi(HA)gam(.)eps(.)p(HA)	10	692.02	5.79	0.022	0.99
psi(HA)gam(T1)eps(T1)p(HA)	12	695.53	9.29	0.0038	1.00
psi(.)gam(.)eps(.)p(.)	4	782.44	96.21	5.0e-22	1.00

The three top models (ΔAICc < 2) were accepted as final and were used to estimate model-averaged probabilities, and the others are given only for comparison. (Psi – initial probability of occupancy, gam – colonization probability, eps – extinction probability, p – detection probability.)

**Fig. 3:**
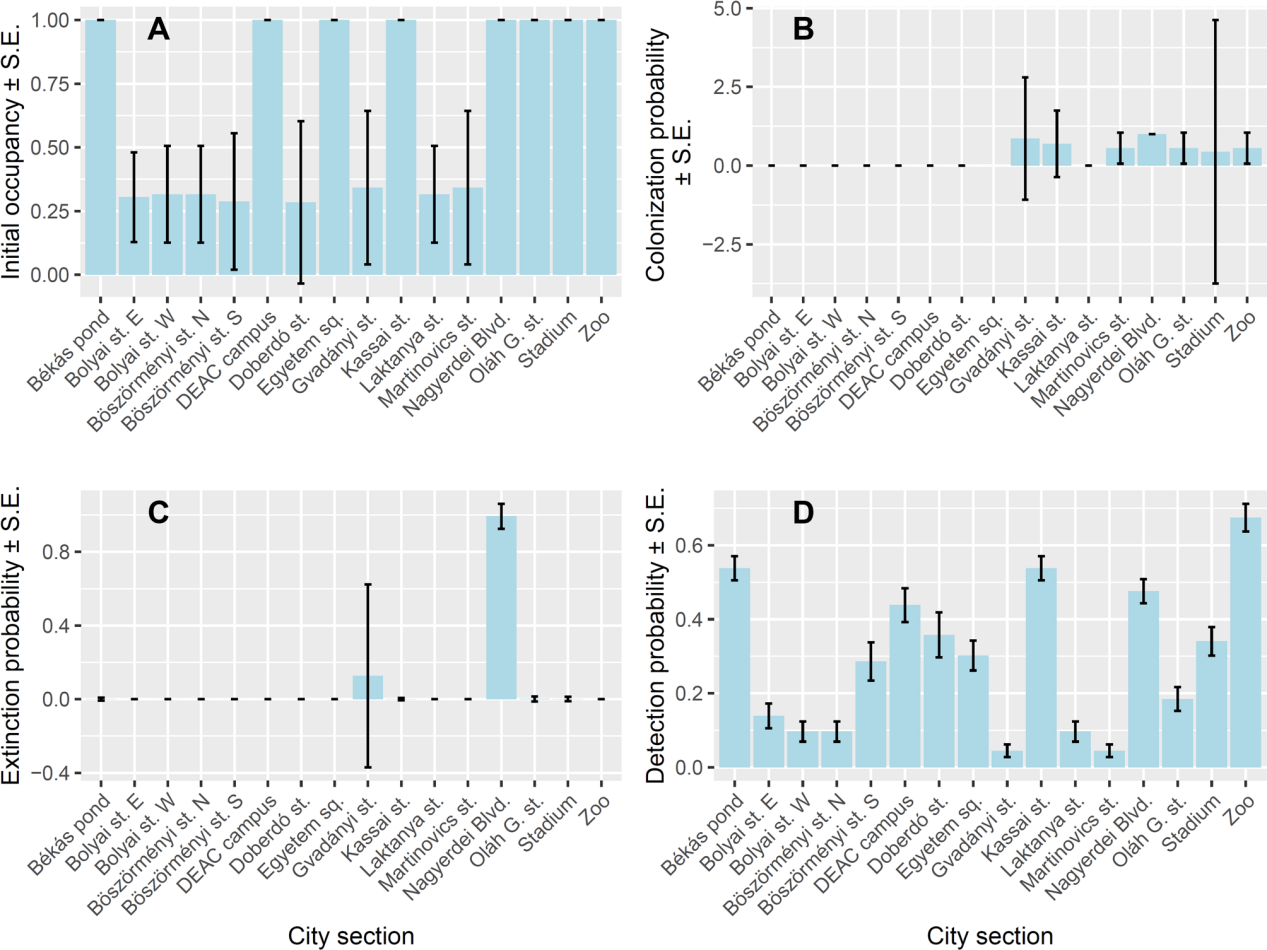
Probabilities of initial occupancy (**A**), colonization (**B**), extinction (**C**) and detection (**D**) of 16 city sections surveyed in Debrecen over three breeding seasons.

Initial occupancy was high (≤ 1) in eight sites and low in eight other sites (Fig. 3A). High initial occupancy was associated with low colonization probability in three sites (Békás pond, DEAC campus, Egyetem Square), and with intermediate colonization probabilities (0.4 < *p* < 0.6) in three sites (Oláh G. st., Stadium, Zoo) (Fig. 3B). Colonization probability was higher (> 0) in sections in the eastern and southeastern sections and close to 0 in the western sections of the study area (Figs. 1 and 3). In the Nagyerdei Blvd. site, initial occupancy, colonization and extinction probabilities were similarly high (Fig. 3C). Extinction probability was low in all other sites (Fig. 3C). Finally, detection probability varied considerably and was lowest in residential areas (Martinovics, Gvadányi, Laktanya, Bolyai streets) (Fig. 3D).

**Fig. 4:**
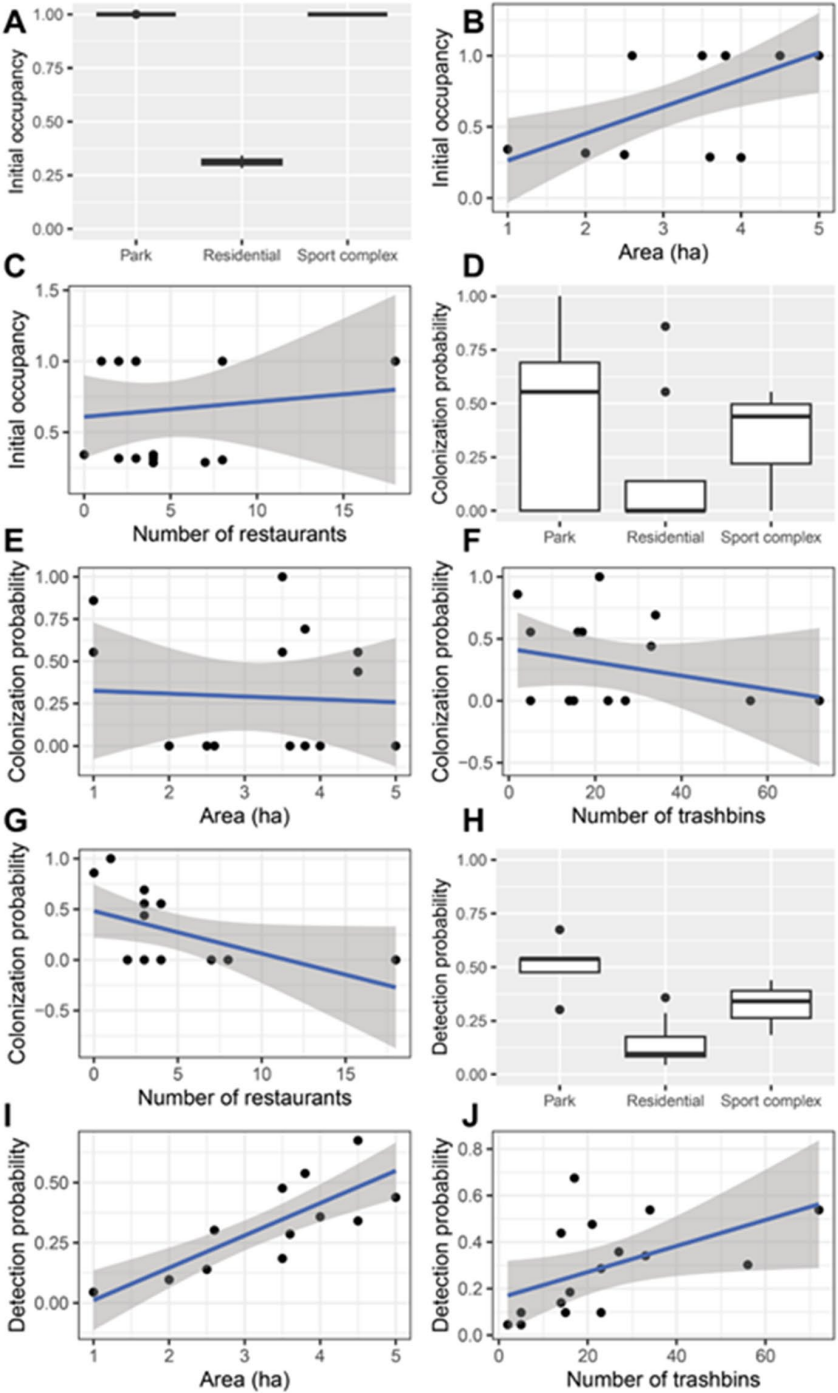
Probabilities of the colonization-extinction dynamics and their relationships with environmental variables in Hooded Crows in 16 sites surveyed in Debrecen over three breeding seasons.

The initial probability of occupancy differed between habitat types (residential < parks = sports complexes) and increased with habitat area and the number of restaurants (Fig. 4A–C; Table 4). Colonization probability also differed between habitat types as it was lower in residential areas than in parks and sports complexes, and it decreased with area and the number of trashbins and restaurants (Fig. 4D–G; Table 4). Extinction probability was not influenced significantly by any of the main effects (n.s., results not shown), whereas detection probability differed between habitat types (again lowest in residential areas), increased with area and the number of trashbins (Fig. 4H–J; Table 4).

**Table 4:** Minimum adequate general linear models testing the effects of habitat type, habitat area, and the number of trashbins, restaurants, and nests in 100-m buffers surrounding 16 surveyed sites on estimated probabilities of initial occupancy, colonization and detection probability (No main effect was significant for extinction probability, these results are not shown).

Dependent variable	Main effects	Coefficient ± S.E.	Test statistic	*p*
Initial occupancy	Habitat type park vs. res	− 0.71 ± 0.005	t = − 134.637	< 0.0001
	Habitat type park vs. spc	0.01 ± 0.006	t = 0.911	0.382
	Area	0.02 ± 0.002	F = 44.020	0.0004
	N restaurants	0.00 ± 0.000	F = 7.195	0.021
Colonization probability	Habitat type park vs. res	− 0.76 ± 0.192	t = − 4.036	0.002
	Habitat type park vs. spc	− 0.32 ± 0.202	t = − 1.597	0.141
	Area	− 0.15 ± 0.074	F = 4.671	0.056
	N trashbins	− 0.01 ± 0.006	F = 11.606	0.007
	N restaurants	− 0.04 ± 0.020	F = 4.651	0.056
Detection probability	Habitat type park vs. res	− 0.21 ± 0.024	t = − 8.570	< 0.0001
	Habitat type park vs. spc	− 0.29 ± 0.026	t = − 11.149	< 0.0001
	Area	0.12 ± 0.010	F = 160.920	< 0.0001
	N trashbins	0.01 ± 0.002	F = 8.283	0.015

## Discussion

We found that the number of crows was higher in sections with more nests in the breeding season at the smaller scale, which could be expected: the more nests, the more crows are likely to be observed in a section. At the larger scale, the number of observed crows increased with the number of trashbins in the breeding season, which supports that anthropogenic food sources may attract Hooded Crows from the section and neighbouring areas.

Outside the breeding season, there were no such clear patterns and crow numbers varied mostly independently of habitat quality. A previous study of wintering crows also found no effect of trashbins on the number of crows, although crow numbers were positively related to park area, water area and length of tram tracks^79^. However, several interactions suggested a temporal change in the spatial distribution of crows in the city. Crow numbers increased at Békás pond, the study section most frequented by humans, as indicated also by the highest number of trashbins and the second highest number of restaurants. At the larger scale, crow numbers decreased in residential areas and in the Zoo during the study, whereas they did not vary considerably in other city sections. Thus, it appears that crows are increasingly using the Békás pond and tend to avoid the Zoo or the residential areas outside the breeding season.

Interestingly, the colonization probability of city sections for breeding was negatively influenced by the number of trashbins and restaurants, which contradicted findings from the repeated-measures analysis that found a positive effect of number of trashbins on crow numbers at the large buffer size. These results suggest that although crows from neighbouring areas may be attracted to certain city sections by the foraging opportunities offered by the high number of trashbins, the crows will also avoid nesting in these areas, likely due to the high human disturbance. This view is further supported by the finding that colonization probability was higher in the calmer eastern-southeastern sections than in the busier western sections of the survey area. Alternative explanations are either that sections with higher number of anthropogenic food sources are frequented by young or non-breeding individuals, or that high intraspecific competition for food or higher predation pressure scares breeding individuals away from these areas. More detailed observations of crow behaviour or data on the space use of marked young and adult individuals could be used to differentiate between these alternative hypotheses.

The number of crows observed per hectare per occasion was highest in two parks (Békás pond, Zoo), but because it was lower in the three remaining parks, the median number was slightly higher in sports complexes than in parks and was lowest in residential areas. Sports complexes probably attract crows by providing large grassy areas for foraging that are similar to grasslands in rural areas. Grasslands in or near zoos are likely central in the colonization of cities by crows^71^ and the proportion of grasslands positively affected the number of Hooded/Carrion Crows counted in a survey of 16 European cities^58^. We found a high variation in crow numbers among the five parks. The two parks with the highest numbers (Békás pond, Zoo) emerged likely because the Zoo area was the first area to be colonized by Hooded Crows in the early 2000s and the food of zoo animals provide a constant source of anthropogenic food to crows, whereas the Békás pond area is the most popular site for recreation with many people and many trashbins and restaurants. Two other parks are on university campuses (Egyetem tér, Kassai St.) and most of the third is an open-air playground (Nagyerdei Blvd.), which have much fewer sources of anthropogenic food than Békás pond and the Zoo. Interestingly, the Nagyerdei Blvd. section had high probabilities of both colonization and extinction, which suggests that there is a high turnover of crows, i.e., many pairs arrive here to breed and many leave. This may be related to the fact that the area lies between the frequented Békás pond and the calmer southeastern residential areas.

We found very few crows in residential areas, which resulted in low probabilities of initial occupancy, colonization and detection in these areas. There were no crows in the four sections along Bolyai and Böszörményi streets, despite abundant green areas (Bolyai St.) or rows of older trees suitable for nesting (Böszörményi St.). This result was unexpected also because some previous studies found no differences in the frequency of the related species *Corvus corone* across urban sectors either in small, medium-sized or large cities^80^. One likely explanation is that both streets have heavy vehicle traffic, and crows are likely to avoid such areas of high disturbance. In four other residential areas with lighter traffic, we found few crows (average 0.02 to 0.17 crows per ha per occasion), suggesting that crows will use residential areas with little disturbance. Nevertheless, the low number of crows in residential areas is promising for urban planning as it suggests that residential areas that have few trashbins and restaurants will not attract large numbers of crows, thus the chances of human-crow conflicts can be minimized.

The Hooded Crow has reached a high degree of synanthropization throughout much of its European range. Numerous studies have documented its progressive colonization of urban areas, characterized by increasing breeding densities in residential zones and the frequent placement of nests near human buildings and infrastructure. Such patterns have been observed in Finland^28^, Slovenia^39^, Hungary^37^, Belarus^38^, and other Central European cities^51,57^. These findings collectively highlight the species’ ecological flexibility and adaptive responses to urban environments, confirming that the Hooded Crow is among the most successful avian urban exploiters in Europe.

Our results support the view that anthropogenic food sources are of utmost importance in the colonization and spread of crow populations in urban environments, a pattern also demonstrated for the Carrion Crow (*Corvus corone*)^41^. Food waste in trashbins, near open-air restaurants etc. offer year-round, near-permanent anthropogenic food sources to crows, although its quality may be lower than that of natural food^81^. While the lower quality of food in cities often leads to lower body size or condition in urban than in rural areas^20^, this may not hold for Hooded Crows because we found no consistent differences in body size between urban and rural areas in a previous study^51^. However, within-city differences may be important, for example, both juveniles and adults were larger in the Zoo than in three other locations in the studied city, indicating better or more sources for feeding in the Zoo and/or that the Zoo is occupied by larger, more dominant birds than other areas^51^.

Wildlife access to food waste is known to exacerbate human-wildlife conflicts, and there is an urgent need to change the way we manage food waste^82^, which needs to be incorporated into urban planning. Hooded Crows are generalist predators, and their distribution and habitat requirements need to be considered in urban planning to reduce human-wildlife conflicts and to minimize the negative effects that crows exert on urban biodiversity^80^. Regional approaches to improving waste management offer the highest chances of success because isolated local solutions are less likely to work^41^. A central measure in such approaches should be a coordinated reduction in food waste and anthropogenic food supply by improved waste management measures such as installation of crow-proof closed-top trashbins in public places or net-covered animal enclosures in zoos^71^. Finally, urban wildlife management targeting the reduction of crow numbers should also benefit from the results of this study. Crow population control can be tricky because crow numbers can increase despite long-term (25 years) increases in the number of crows culled in hunting activities^41^. Our study can improve the efficiency of crow control measures as it identified city sectors where crow control by trapping could be most efficient both in the breeding season, i.e., in residential areas with relatively high colonization probability, and outside the breeding season, i.e., in the Békás pond and the Zoo area, where crows aggregate during the winter.

In conclusion, our study supports the view that the availability of anthropogenic food sources has primary importance in the habitat use of Hooded Crows within a city, and that habitat type and area are of secondary importance. Our results suggest that although crows are attracted to certain city sections by the foraging opportunities offered by anthropogenic food sources outside the breeding season, crows will avoid nesting in these areas, likely due to high human disturbance. The low number of crows in residential areas is promising for urban planning as it suggests that residential areas that have few trashbins and restaurants will not attract large numbers of crows, thus the chances of human-crow conflicts can be minimized. Our study also suggests that improved management of food waste by installation of closed-top trashbins in public places or net-covered animal enclosures in zoos, may further reduce the availability of anthropogenic food sources to crows. Finally, our study suggests that crow control measures by trapping are most likely to be successful in residential areas in the breeding season and in areas with high anthropogenic food availability outside the breeding season. We believe that this knowledge will be useful in urban planning, urban wildlife management and the conservation of urban biodiversity.

## Supplementary Information

Below is the link to the electronic supplementary material.

Supplementary Material 1

## Data Availability

All data used in the analyses are available from Zenodo at 10.5281/zenodo.18506567.
