# Supplementary material for: Spatial and temporal dynamics in the use of urban habitats by Hooded Crows
Source: Sci Rep. 2026 Feb 19;16:9881. doi: 10.1038/s41598-026-40561-z (PMC13018644; doi:10.1038/s41598-026-40561-z)
Supplement: Supplementary file 1 — Supplementary Material 1 [file 41598_2026_40561_MOESM1_ESM.pdf]

SUPPLEMENTARY MATERIAL

To accompany “Spatial and temporal dynamics in the use of urban habitats by Hooded Crows” by Paládi et al. in Scientific Reports

Authors:
Petra PALÁDI; Isma BENMAZOUZ; Máté TÓTH; László KÖVÉR; Szabolcs LENGYEL

Corresponding author:
Petra PALÁDI, paladi.petra@agr.unideb.hu
[1]HUN-REN Centre for Ecological Research, Institute of Aquatic Ecology, Conservation Ecology Research Group, Bem tér 18/c, 4026, Debrecen, Hungary
[2] University of Debrecen, Department of Nature Conservation, Zoology and Wildlife Management, Böszörményi út 138., 4032, Debrecen, Hungary

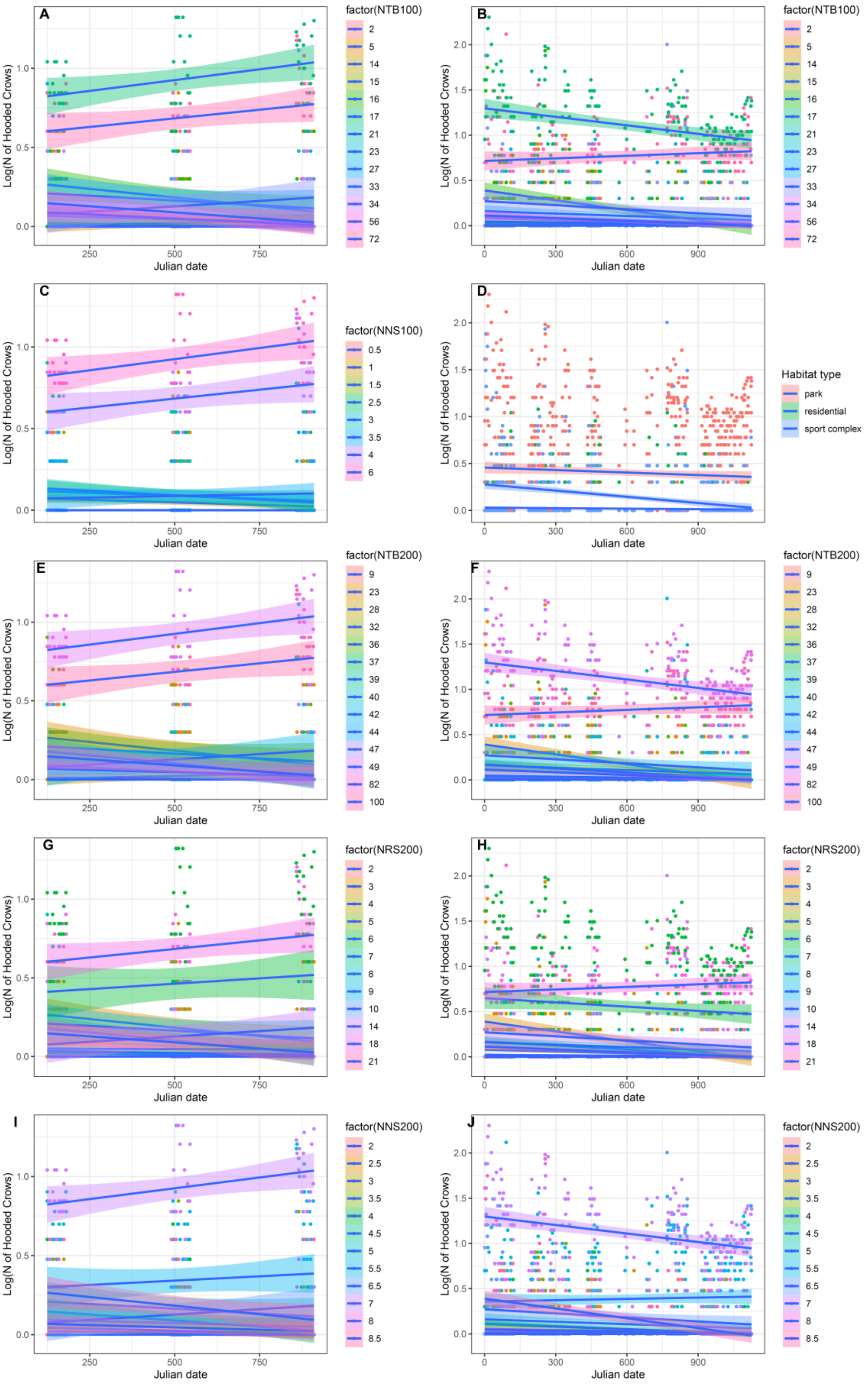

Fig S1 Interaction plots between either the between-subject effect (habitat type) or the covariates (number of trashbins, restaurants, nests) and Julian date. Lines were fitted and continuous covariates were treated as factors for visual guidance only for this graph (NTB – number of trashbins, NRS – number of restaurants, NNS – number of nests; numbers 100 and 200 indicate buffer size)
